# Mucosal Adjuvant Potential of *Quillaja* saponins and Cross-linked Dextran Microspheres, Co-administered with Liposomes Encapsulated with Tetanus Toxoid

**Published:** 2012

**Authors:** Faezeh Moghadam Ariaee, Mohsen Tafaghodi

**Affiliations:** a *School of Pharmacy, Mashhad University of Medical Sciences, Mashhad, Iran.*; b *Pharmaceutical Research Center, Mashhad University of Medical Sciences, Mashhad, Iran.*

**Keywords:** Nasal immunization, Tetanus toxoid, *Quillaja *saponin, Liposomes, Cross-linked, Dextran microspheres

## Abstract

Intranasal vaccination is particularly a striking route for mucosal immunization, due to the ease of administration and the induction of both mucosal and humoral immunity. However, soluble antigens (Ag) are not sufficiently taken up after the nasal administration and need to be co-administered with adjuvants, penetration enhancers or encapsulated in particles. So, in this study, tetanus toxoid (TT) as a model Ag was entrapped in nonionic liposomes. The effect of the co-administration of *Quillaja *saponin (QS) as an adjuvant and cross-linked dextran microspheres (CDM) as penetration enhancer on immune responses was also studied. TT or TT + QS loaded liposomes were prepared by dehydration-rehydration method (DRV), followed by the extrusion through 400 nm filters. Some formulations were mixed with CDM. Liposomes were first characterized for their size range, mean diameter and morphology using particle size analyzer, optical and transmission electron microscopes. The volume mean diameter of liposomes was determined as 3836 ± 179 and 624 ± 114 nm before and after the extrusion, respectively. Structural efficiency of TT extracted from liposomes was confirmed by SDS-PAGE method. Encapsulation efficiencies of TT and QS were 44 ± 8.50% and 60 ± 6.02%, respectively. Rabbits were nasally immunized with various formulations and serum IgG titers and nasal lavage sIgA titers were determined by an ELISA method. TT + QS liposomes induced higher sIgA levels in comparison with TT liposomes (p < 0.05), but the difference in serum IgG levels was not significant. Results indicated that neutral liposomes administered nasally, have a good potential for induction of mucosal immunity and co-encapsulation of QS and TT in liposomes improved the systemic and mucosal immune responses.

## Introduction

The present vaccines administered parenterally, provide only systemic antibodies that do not necessarily reach mucosal surfaces. Therefore, immunization via mucosal routes has emerged as a rising interest in recent years (1, 2). Since the protection against the pathogenic organisms is more related to the presence of antibody in local secretions than to serum antibody (3), the prominent goal of mucosal vaccination is the production of local antibodies at the sites where pathogens enter the body (4). Among various routes for induction of mucosal immunity to improve the defense against the infections, intranasal (IN) immunization is a highly efficient alternative for parenteral vaccination. IN immunization by particulate antigens could target them to nasal-associated lymphoid tissue (NALT) and subsequently produce serum antibodies as well as the secretory immunoglobulin A (sIgA) on the mucosal surfaces (2, 5). Thus, nasal delivery of vaccines serves as an important route of protecting the upper respiratory tract from the infections, as well as diseases involving other mucosal surfaces (6). In comparison to oral immunization, IN immunization generally needs much smaller doses which is important for many recombinant antigens that are often costly (7).

The first determinant step in the success of vaccines to induce the mucosal and systemic immunity, is the development of efficient delivery systems to present antigens to mucosal surfaces (8). Particulate antigens exercise more effectively than soluble antigens; probably due to the more efficient endocytosis of these antigens by mucosal-associated lymphoid tissue (MALT) M-cells (9). Liposomes as the carrier and particulate antigen delivery systems could be used to potentiate the immune responses against the encapsulate antigens. It has been shown that liposomes could increase the interaction of soluble antigens with antigen presenting cells (APCs) and macrophages after being uptaken by M-cells (10).

There are some physicochemical factors affecting the immunostimulatory potential of liposomes including Tc, size, phospholipid composition and surface charge of liposomes as well as other factors such as antigen characteristics and the administration conditions (11, 12). In addition, some strategies have also been suggested to elevate the immune stimulation potential of liposomes, like the co-administration of liposomes with different adjuvants (13).

Saponins are an extremely variant group of glycosides that are found mostly in plants (14). *Quillaja *saponins (QS), extracted from *Quillaja *saponaria bark, have been confirmed to have immunoadjuvant effect in several studies. The purified fraction of *Quillaja *saponins (Quil A) is currently approved for the veterinary vaccines and these are commercially available vaccines adjuvanted with QS, like bovine respiratory syncytial virus vaccine (15, 16). Further purification of this fraction leads to the production of more small parts including QH-A, QH-C and the mixture Iscoprep ^TM^ 703 and QS-21 or Stimulon ^TM^ (17). Among these, QS-21 has been evaluated extensively as an adjuvant for human vaccines with clinical trials ongoing and fulfilled for a number of diseases including melanoma, influenza, herpes simplex virus and HIV-1 (16, 18).

Mucoadhesive polymers, also named as the absorption enhancer adjuvants, can decelerate the nasal clearance and enhance the residence time between the liposomes and nasal tissues (19, 20). Among them, cross-linked dextran microspheres (CDM, Sephadex®) have been shown helpful to overcome the mucosal barriers by various mechanisms (19, 21). These microspheres have also been investigated for encapsulation of liposomes (22).

At the present study, neutral tetanus toxoid (TT)-containing liposomes were prepared by dehydration-rehydration method. The stability of encapsulated antigen was studied via the SDS-PAGE method. After nasal immunization in rabbits, the immunoadjuvant potential of liposomes, CDM microspheres and Quillaja saponins in the induction of systemic and mucosal immune responses were evaluated by determination of the sera IgG and nasal lavage sIgA titers.

## Experimental

Phosphatidylcholine from egg yolk (Egg PC) was purchased from Fluka (Buchs, Switzerland) and cholesterol (Chol) was obtained from Merck (Darmstadt, Germany). purified Quillaja saponin (QS) was obtained from Sigma (St Louis, USA). Cross-linked dextran microsphere (Sephadex^®^ G-150) was purchased from BioGene (Sweden). Tetanus toxoid (TT) solution (1700 Lf/mL) was from Razi Inc. (Hesarak, Iran). Anti-rabbit IgG and IgA antibodies were from Sigma (Missouri, USA) and Bethyl Laboratories Inc. (Texas, USA), respectively. White albino rabbits weighing 1.4-2.3 Kg were provided by Razi Inc. (Hesarak, Iran).

*Preparation of liposomes encapsulated with TT and/or QS*

Liposomes composed of PC and Chol (16.5 μmol/mL from each one), were prepared as dehydration-rehydration vesicles (DRV) (23). Briefly, the lipid phase was dissolved in chloroform: methanol; 2:1 v/v, in a round-bottom flask. The solvent removal was done by rotary evaporation method. To ensure the total removal of the solvent, the lipid film was then freeze-dried (Heto Drywinner, DW3, Heto-Halter, Allerod, Denmark) for 1 h. The lipid film was hydrated with distilled water and vortexed at 45° C for 30 min. The consequent multilamellar vesicles (MLVs) were turned to small unilamellar vesicles (SUVs) using bath sonicator (Kerry, UK) for 10 min. The resulting SUVs were mixed with encapsulated material (TT and QS). Dehydration of these SUVs was performed by the flash freezing in dry ice-acetone and freeze-drying overnight. The dried broken liposome powder was rehydrated at room temperature for 1 min, by gentle vortexing with a distilled water volume equivalent to one tenth of the final volume (100 μL). Thirty min later, the liposomes were diluted with PBS (phosphate-buffered saline) to 1 mL as final volume. The later procedure was the separation of supernatant by centrifugation at 13000 g for 15 min. The dilution and separation of supernatant was repeated thrice. The final multilamellar vesicles (MLVs) prepared by dehydration-rehydration method were 11 times passed through the extruder (Avestine, Canada) equipped with 1000, 400 nm polycarbonate filters, respectively (24).

*Morphology and particle size analysis*

The optical microscope (Olympus, Japan) was used for the morphological studying of liposomes. The transmission Electron Microscope (Leo, UK) was also used for studying the vesicles, after staining with 2% uranyl acetate. The volume mean diameters of liposomes before and after the extrusion were determined by a laser diffraction size analyzer (Klotz, Germany).

*Encapsulation efficiency of tetanus toxoid (TT)*

Encapsulation efficiency was determined with an indirect method by determination of non-entrapped TT in the supernatant of liposomal suspensions. The amount of TT in supernatants was determined by Bradford protein assay method (25).

*Structural stability of encapsulated TT*

The structural stability of encapsulated TT was evaluated by sodium dodecyl sulfate-polyacrylamide gel electrophoresis (SDS-PAGE) method. Four samples including 2 liposome suspensions before and after the extrusion, original TT and a molecular weight reference marker were loaded on a 10% acrylamide gel. Protein bands were visualized by silver nitrate staining (26).

*Nasal immunization studies*

White albino rabbits weighing 1.4-2.3 Kg (four animals per group) were nasally immunized with the following formulations in days 0 and 14.

(1) 40 Lf TT solution

(2) 40 Lf TT encapsulated in liposomes, (TT) liposomes (suspension)

(3) 40 Lf TT + 20 μg QS encapsulated in liposomes, (TT + QS) liposomes (suspension)

(4) 40 Lf TT + 10 mg CDM + liposomes (mixed and freeze-dried), TT + CDM + liposomes (powder)

(5) 40 Lf TT encapsulated with liposomes + 10 mg CDM (mixed and freeze-dried), (TT) liposomes + CDM (powder)

(6) 40 Lf TT + 20 μg QS encapsulated in liposomes + 10 mg CDM (mixed and freeze-dried), (TT + QS) liposomes + CDM (powder)

Two hundred μL of liposomal suspension (100 μL in each nostril) or its equivalent powder was employed for each administration. Suspension and powder forms were administered using an automatic pipette and a thin polyethylene tube attached to a syringe, respectively. Each animal was bled in day 21. After bleeding, the nasal cavity was washed with 5 mL sterile PBS buffer. Sera and nasal lavages of each group were pooled and kept frozen until immunological assays.

*Determination of serum anti-TT IgG titers and nasal lavage anti-TT IgA titers*

Anti-TT antibodies in the rabbit sera and nasal lavages were determined by an ELISA method (27). IgG antibodies in the rabbit serum were determined by end-point titration. End- point titers for IgG were defined as the highest serum dilution that resulted in an absorbance value (OD 450) equal to normal serum. In the case of sIgA, absorbance values (OD 450 nm) were used. 

*Statistical analysis*

Statistical analysis was carried out one-way ANOVA and unpaired Student’s t-test. P-values less than 0.05 were regarded as significant. 

*Ethics in animal investigations*

The protocols of animal studies were approved by regional ethics committee. 

## Results

*Morphology and size of liposomes*

**Figure 1:**
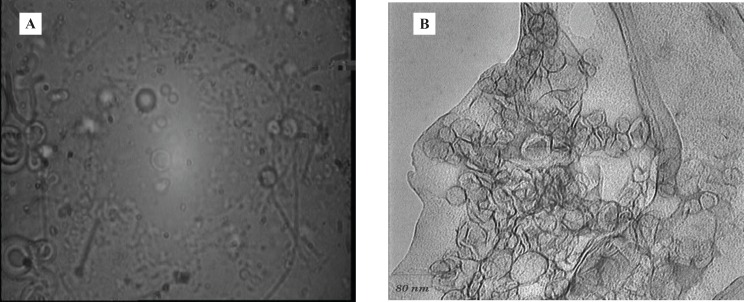
(A) Liposomes under optical (B) and transmission electron microscopes

Under the optical and also Transmission Electron microscope, multilamellar morphology was observed for the liposomes (Figure 1). 

**Figure 2:**
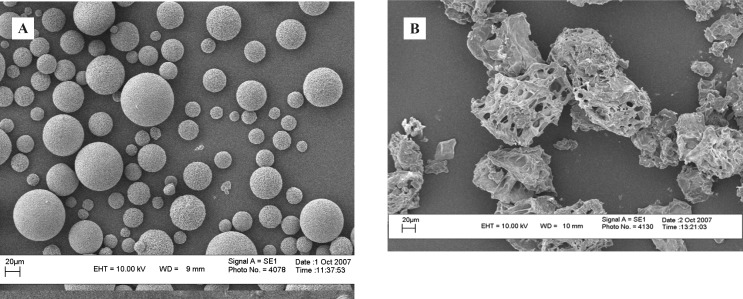
CDM microspheres before (A) and after (B) freeze-drying

Using particle size analyzer, volume mean diameter of liposomes was determined as 3836 ± 179 and 624 ± 114 nm, before and after extrusion respectively (n = 3). The mean diameter of CDM microspheres was determined by direct measurement of 100 microspheres under the optical microscope, equipped with an eyepiece reticule, as 17.4 ± 9.4 μm (Figure 2).

*Encapsulation efficiency of TT and QS in liposomes*

The encapsulation efficiency of TT and QS in liposomes was found to be 44 ± 8.50% and 60 ± 6.02%, respectively (n = 3).

*Structural stability of encapsulated TT*

**Figure 3:**
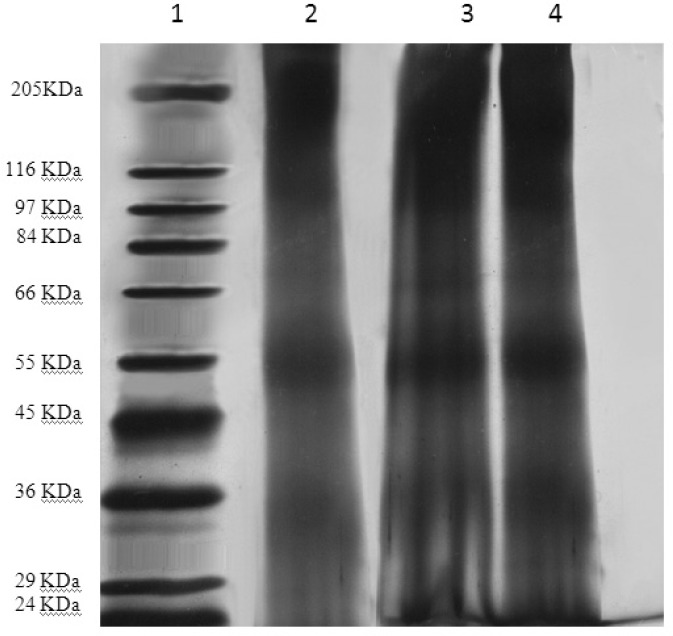
The SDS-PAGE gel Samples were loaded onto a 10% acrylamide gel. Protein bands were visualized by silver nitrate staining. Samples from left to right: 1. molecular weight reference marker; 2. original TT solution; 3 and 4. liposomal suspensions before and after extrusion.

By SDS-PAGE method, the identical bands were observed for both encapsulated and original TT (Figure 3). This observation proves the stability of tetanus toxoid structure in the liposome preparation and extrusion processes.

*Serum anti-TT IgG titers*

**Figure 4:**
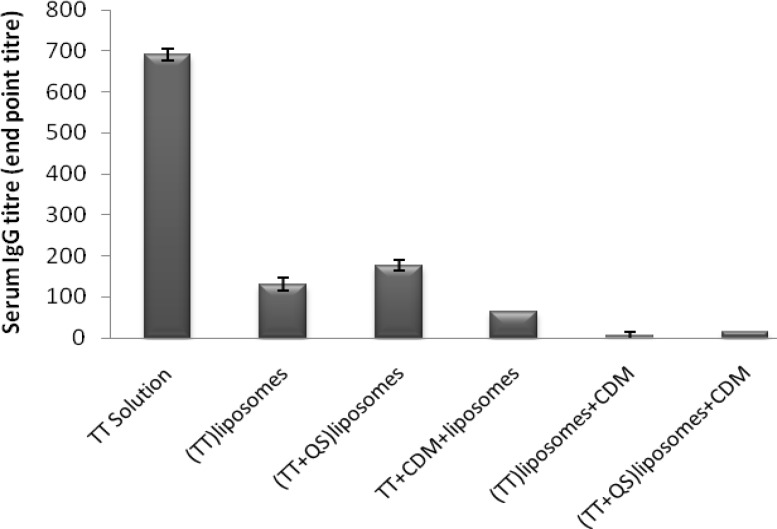
Serum anti-TT IgG titers (mean ± SE). Rabbits (n = 4) were nasally immunized with 40 Lf TT and 20 μg QS with or without CDM, at weeks 0 and 2 and were bled at week 3. Sera anti-TT IgG titers (end-point titration) were determined by an ELISA method

Co-encapsulation of TT and QS within liposomes including (TT + QS) liposomes (suspension) and (TT + QS) liposomes + CDM (powder) improved IgG titers compared to their matching groups without saponin but the difference was not significant (p > 0.05). Among the groups immunized with liposomal formulations, the highest IgG titers were seen in group immunized with (TT + QS) liposomes. However, systemic response in group immunized with TT solution was significantiy higher than all of those received liposomal formulations (p < 0.001) (Figure 4).

*Nasal lavage anti-TT sIgA titers*

**Figure 5:**
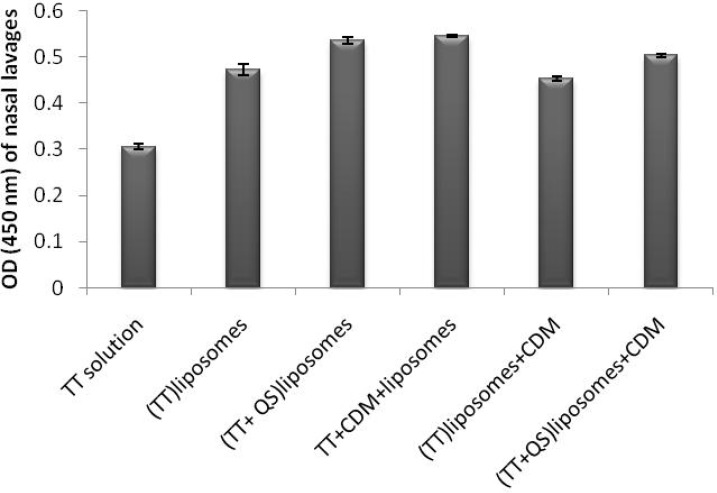
Nasal lavage anti-TT sIgA titers. Rabbits (n = 4) were nasally immunized with 40 Lf TT and 20 μg QS with or without CDM, at weeks 0 and 2 and nasal lavages were collected at week 3. Lavages were pooled and Anti-TT IgA titers (absorbance) were determined by an ELISA method

SIgA titers in all groups were significantly higher than the control group (p < 0.01). Co-encapsulation of TT and QS within liposomes including (TT + QS) liposomes (suspension) and (TT + QS) liposomes + CDM (powder) could significantly increase the mucosal sIgA titers compared to their counterpart groups without saponin (p < 0.05 and p < 0.01, respectively). Among the groups immunized with various formulations, the highest mucosal sIgA titers were related to animals immunized with (TT + QS) liposomes and with TT + CDM + liposomes (p < 0.05) (Figure 5).

## Discussion

Immunization studies indicated that co-encapsulation of *Quillaja *saponin (QS) and TT within the neutral liposomes could significantly improve the mucosal sIgA titers (p < 0.05) and also improved the systemic IgG titers (although differences were not significant) compared to the encapsulation of TT alone. However, cross-linked dextran microspheres (CDM) failed to improve immune responses.

Particulate antigens could be taken up by microfold cells (M-cells) in the NALT (28). Reduced systemic immune responses have been attributed to the restricted distribution (due to the size of particulate systems especially over 5 μm) of particulate antigens to achieve the cervical lymph nodes (28, 29). In contrast, the uptaken antigen can induce a local (and also a far) mucosal immune response or lead to tolerance (30). This is in line with our findings from the present and previous studies after nasal administration of liposomes; whereas, the liposomal formulation induced high mucosal sIgA titers; the systemic responses were even lower than that of TT solution (26, 31, 32). Allen *et al. *have stated that drug delivery by small liposomes is more efficient than that with larger liposomes, most probably because of more effective internalization (33). Our extruded liposomes had a mean diameter of 624 ± 114 nm but it appears that even this size of liposomes principally remain in the IgA inductive sites and are not able to translocate to regional lymph nodes, which is mandatory for the induction of systemic immune responses. However, they are proper for the induction of mucosal immune responses (34). It has been also shown in our previous gamma-scintigraphic study that the neutral liposomes are potentially high mucoadhesive and after a 4 h follow up, only 18 ± 2.9% of administered liposomes were cleared from the human nose (35). This point gives the neutral liposomes adequate time to interact with IgA inductive sites at nasal mucosa and stimulate the high mucosal response, but does not increase the chance of penetration into the M-cells of NALT and induction of sufficient systemic responses (26). Neutral liposomes have neither membrane toxicity, nor local irritation in human nose as studied in our previous study. This observation proved them as a preferential option for human administration (26).

Quil A is a purified saponin extracted from the bark of *Quillaja *saponaria (36). QS saponins have a confirmed immunoadjuvant potential but also accompanied by an intrinsic hemolytic activity (37). Some studies have demonstrated that the optimization of saponin formulation could be achieved by introducing it within liposomes. This strategy declines lytic activity without destroying its adjuvanticity (38, 39). The mechanism of the action of QS is not completely elucidated, although *in-vitro *studies suggest that QS could optimize T-cell response via the promotion of antigen presentation by APC. Likewise, it has been confirmed that QS21 improve the B-cell response while still unclear whether it is either through a direct effect or via APC/T-cell stimulation (40).

QS21 has been shown in healthy volunteers to elevate both humoral and cell-mediated immunity against the recombinant antigens or peptides when given with parenteral vaccines (41-43). In mice, QS21 plays as an effective adjuvant for CTL induction and promotes the secretion of Th1 cytokines including IL-2 and IFN-γ and also IgG2a antibodies (37). Lately, the orally administered QS-21 has been reported to reveal the adjuvanticity for the induction of both systemic and mucosal immunity to co-administered TT. An optimal dose (*i.e. *50 mg) of oral QS-21 enhanced the systemic response including TT-specific IgG1 and IgG2b antibodies as well as mucosal sIgA responses (44). Co-encapsulation of TT and QS within liposomes including (TT + QS) liposomes (suspension) and (TT + QS) liposomes + CDM (powder) could significantly increase the mucosal sIgA titers compared to their counterpart groups without saponin (p < 0.05 and p < 0.01, respectively), but the increase in the serum IgG titers was not significant (p > 0.05). In our another study, Co-encapsulation of TT and QS within the PLGA nanospheres could increase the serum IgG and mucosal sIgA titers compared to the nanospheres encapsulated with TT alone (45). These results confirmed that using carriers for the co-delivery of antigen (TT) and adjuvant (QS) to the same APCs is a key parameter in the improvement of immune responses. The difference observed between PLGA nanospheres and liposomes could be rooted from their surface charge, rigidity of particles or the mucoadhesive potential.

Pre-formed cross-linked dextran (Sephadex®) microspheres have been studied for the delivery of peptides and proteins via nasal rout (46, 47), but as a carrier for the nasal vaccination, they have induced little or no immune responses (48, 49). CDM microspheres are too big to be taken up by M-cells or penetrate through the epithelial barrier, but they act as a penetration enhancer (49). In our previous study, TT was co-administered with CDM or CpG ODN adjuvant. CDM induced comparable systemic IgG titers as CpG ODN (p > 0.05) (21).

Moreover, our previous gamma-scintigraphic study on human nose showed that after 4 h, about 27% of nasally administered CDM microspheres were still remained in the nasal cavity (19) which confirms the mucoadhesion potential of these microspheres.

Other mechanisms have also been proposed for the absorption enhancement effects of CDM, including: reversible shrinkage of the epithelial cells by absorption of water and consequently expanding the tight junctions (50) and providing a local high drug concentration in close contact with the epithelial surface due to the gel system formation (51).

Mucosal immune responses obtained from the CDM-containing formulations were similar to their counterpart formulations without CDM (p > 0.05). As the efficient interaction of antigen with NALT has an important role in the stimulation of sIgA titers, the lower sIgA titer induced with CDM formulations could be attributed to the size of microspheres and their disability in interaction with NALT (21). Moreover, reduced systemic responses with neutral liposomes, as expressed above, were further suppressed in CDM-containing formulations. CDM microspheres were used in the form of powder formulations since in contrast to nasal solutions, dextran and starch microspheres as powders will be deposited in the anterior part of the nose, with no or few mucociliary clearance (51). Therefore, it was expected that these microspheres could be retained for more extended period of time than that of solution dosage forms. However, it seems that due to the low ability of neutral liposomes to interact with M-cells, obviously neither the mucoadhesion potential, nor the penetration enhancing effect of CDM could overcome this limited interaction. Another explanation for poor results observed with formulations administered with CDM was the difficulty in their administration. CDM, when mixed with liposomes and also after the freeze-drying, showed poor flow and could not be administered as precise as suspensions.

## Conclusion

The collective results indicated that the neutral liposomes administered nasally, have a good potential for induction of mucosal immune responses. Co-encapsulation of *Quillaja *saponins resulted in higher systemic and mucosal immune responses, although difference in serum IgG levels was not statistically significant. Co-administration of liposomes with cross-linked dextran microspheres failed to enhance the mucosal and systemic responses.
